# Supplementary material for: Oscillations support short latency co-firing of neurons during human episodic memory formation
Source: eLife. 2022 Nov 30;11:e78109. doi: 10.7554/eLife.78109 (PMC9731574; doi:10.7554/eLife.78109)
Supplement: Figure 6—source data 1. [file elife-78109-fig6-data1.docx]

| **Figure 6 – source data 1: Regions where distally and locally coupled neurons were recorded** | | |
| --- | --- | --- |
| **Patient IDs** | **Distally coupled (up-stream)** | **Locally coupled (down-stream)** |
| P04 | left mid. Hipp. MW4 N1 SU | left EC MW3 N1 MU |
| P04 | left EC MW8 N1 SU | left mid. Hipp. MW4 N1 SU |
| P04 | left mid. Hipp. MW7 N1 MU | left EC MW3 N1 MU |
| P05 | left mid. Hipp. MW1 N1 MU | right mid. Hipp. MW3 N1 MU |
| P05 | left mid. Hipp. MW5 N2 MU | right mid. Hipp. MW3 N1 MU |
| P07 | left mid. Hipp. MW8 N1 SU | right ant. Hipp. MW6 N1 MU |
| P07 | right mid. Hipp. MW4 N1 SU | right ant. Hipp. MW6 N1 MU |
| P07 | right ant. Hipp. MW4 N1 MU | right mid. Hipp. MW2 N2 MU |
| P07 | right ant. Hipp. MW7 N1 MU | right mid. Hipp. MW2 N2 MU |
| P07 | right mid. Hipp. MW3 N2 SU | right ant. Hipp. MW7 N2 SU |
| P07 | right mid. Hipp. MW4 N1 MU | right ant. Hipp. MW7 N2 SU |
| P08 | left Amy. MW1 N1 MU | right mid. Hipp. MW8 N1 SU |
| P08 | left Amy. MW3 N1 MU | right mid. Hipp. MW8 N1 SU |
| P08 | left Amy. MW5 N1 SU | right mid. Hipp. MW8 N1 SU |
| P08 | left Amy. MW8 N1 MU | right mid. Hipp. MW8 N1 SU |
| P08 | left Amy. MW3 N1 MU | right mid. Hipp. MW2 N1 SU |
| P08 | left Amy. MW3 N1 MU | right mid. Hipp. MW1 N1 MU |
| P08 | right mid. Hipp. MW1 N1 MU | left Amy. MW3 N1 MU |
| P08 | right mid. Hipp. MW1 N1 MU | left Amy. MW8 N1 MU |
| P09 | left ant. Hipp. MW7 N1 MU | right mid. Hipp. MW6 N1 SU |
| P09 | left PHC MW6 N1 MU | right mid. Hipp. MW6 N1 SU |
| P09 | right post. Hipp. MW2 N1 MU | right mid. Hipp. MW6 N1 SU |
| P09 | right post. Hipp. MW5 N1 MU | right mid. Hipp. MW6 N1 SU |
| P09 | left PHC MW5 N1 MU | right mid. Hipp. MW4 N1 MU |
| EC = entorhinal Cortex; ant. = anterior; mid = middle; post. = posterior; Amy. = Amygdala; Hipp. = Hippocampus; PHC = parahippocampal cortex; MW = Microwire on which neuron was recorded; N = cluster ID as obtained by WaveClus ; SU = Single Unit; MU=Multi Unit | | |
